# Prognostic Factors for Elderly Patients Treated With Stereotactic Body Radiation Therapy for Pancreatic Adenocarcinoma

**DOI:** 10.3389/fonc.2018.00282

**Published:** 2018-07-27

**Authors:** Philip A. Sutera, Mark E. Bernard, Hong Wang, Dwight E. Heron

**Affiliations:** ^1^Department of Radiation Oncology, UPMC Hillman Cancer Center, University of Pittsburgh School of Medicine, Pittsburgh, PA, United States; ^2^Department of Radiation Medicine, University of Kentucky, Lexington, KY, United States; ^3^Department of Biostatistics, University of Pittsburgh, Pittsburgh, PA, United States

**Keywords:** pancreatic cancer, elderly population, stereotactic body radiation therapy, prognostic factors, radiation toxicity

## Abstract

**Introduction:** Pancreatic ductal adenocarcinoma (PDAC) commonly presents later in life with a median age at diagnosis of 70 years. Unfortunately, elderly patients are significantly underrepresented in clinical trials. Stereotactic body radiation therapy (SBRT) is a promising treatment modality in this population as it has demonstrated excellent local control with minimal toxicity. We aimed to determine prognostic factors associated with outcomes in elderly patients treated with SBRT.

**Materials and Methods:** Elderly patients older than 70 treated with SBRT for PDAC at our institution, from 2004 to 2014 were included. Our primary endpoints included overall survival (OS) and local-progression-free survival (LPFS). Secondary endpoints included regional-progression-free survival (RPFS), distant-progression-free-survival (DPFS) and radiation toxicity. Endpoints were analyzed with the Kaplan-Meier method. The association of these survival endpoints with risk factors was studied with Cox proportional hazards models.

**Results:** We identified 145 patients with 146 lesions of pancreatic adenocarcinoma with a median age at diagnosis of 79 (range, 70.1–90.3). SBRT was delivered to a median dose of 36 Gy (IQR 24–36). Surgical resection was performed on 33.8% of the total patients. Median follow-up was 12.3 months (IQR 6.0–23.3 months) and the median survival for the entire cohort 14.0 months with a 2-year OS of 27%. Multivariate analysis (MVA) demonstrated surgery [*p* ≤ 0.0001, HR 0.29 (95% CI, 0.16–0.51)] and post-SBRT CA19-9 [*p* = 0.009, HR 1.0004 (95% CI, 1.0002–1.0005)] significantly associated with overall survival. Recurrent lesions [*p* = 0.0069, HR 5.1 (95% CI, 1.56–16.64)] and post-SBRT CA19-9 levels [*p* = 0.0107, HR 1.0005 (95% CI, 1.0001–1.0008)] were significantly associated with local control on MVA. For the entire cohort, 4.1% experienced acute grade 2+ toxicity, and 2% experienced late grade 2+ toxicity at 2 years.

**Conclusion:** This review demonstrates prognostic factors in elderly patients with PDAC treated with SBRT. We identified surgical resection and post-SBRT CA 19-9 as predictive of overall survival in this population. Additionally, we show low acute and late toxicity following SBRT in elderly patients.

## Introduction

Pancreatic adenocarcinoma is the 11th most common cause of new cancer cases, but is the third leading cause of cancer mortality in the United States. Despite aggressive multidisciplinary efforts in recent years, the 5-year mortality remains dismal at 10–30% depending on the resectable status (1–3). Currently, surgical resection remains the most significant prognostic factor, with adjuvant chemotherapy playing and important supportive role. Notably, elderly patients make up a considerable proportion of those with pancreatic cancer with a median age at diagnosis of 70 years and median age at death of 72 years (4). Unfortunately, although elderly patients account for half of this population, they are significantly underrepresented in clinical trials meant to guide treatment decisions (5). This underrepresentation makes it difficult to extrapolate the role of various treatment strategies for this specific population.

Elderly patients with pancreatic adenocarcinoma present a myriad of challenges making it difficult to administer aggressive multi-modality treatment. Standard treatment with invasive surgical procedures and multi-agent chemotherapy may not be tolerable in frail patients with significant comorbidities (6, 7). Additionally, traditional radiation therapy with 6 weeks of external beam radiation therapy (EBRT) has been associated with significant treatment-related morbidity (8). Stereotactic body radiation therapy (SBRT) was developed to deliver a high dose of radiation therapy in few treatments while minimizing dose to surrounding tissue (9). This method has demonstrated excellent local control with minimal toxicity in a variety of diseases (10, 11). Herein we aimed to determine outcomes in elderly patients treated with SBRT for pancreatic adenocarcinoma.

## Methods

### Patient population

In accordance with our institutional review board, elderly patients (age >70) with histologically-proven pancreatic adenocarcinoma between 2004 and 2014 were reviewed. Patients with resectable, borderline resectable, unresectable, medically-inoperable, and recurrent tumors were included in this study. Patients were excluded if they had distant metastasis at diagnosis. SBRT was performed using either a CyberKnife® robotic radiosurgery (Accuray Inc., Sunnyvale, CA) or non-robotic linear accelerator based platforms (Trilogy® or TrueBeam®) (Varian Medical Systems, Palo Alto, CA). Patient variables included were age, race, gender, surgical status, chemotherapy treatment, prior EBRT, and SBRT dose, dosimetry, and toxicity were collected.

### Definition of parameters

Resectable status was determined by a multidisciplinary case review using National Comprehensive Cancer Network (NCCN) guidelines for resectable, borderline resectable, and unresectable disease. Prophylactic proton-pump inhibitors was not routinely recommended for patients. Local, regional, and distant progression were determined based on radiographic findings on follow up and/or confirmatory biopsy if done. Local progression was identified as progressive disease (PD) using RECIST 1.1 criteria which is characterized by at least a 20% increase in the sum of diameters of the tumor and a minimum of a 5 mm increase (12). Regional failure was defined as disease progression to the regional nodes defined as n1, n2, or n3 by the Japanese Pancreas Society (JPS) classification (13, 14) (or new tumor growth within the pancreas outside of the radiation field). Toxicity was graded retroactively with the Common Terminology Criteria for Adverse Events Version 4.0 (CTCAE 4.0). Patients included in this review were simulated in the supine position using four-dimensional CT-scan, utilizing 1.25 mm slices, with IV contrast in a vacuum lock bag and wingboard. The GTV was determined based on the simulation CT scan and diagnostic CT scans. The PTV margin was added to be ~3 mm from GTV. When the GTV or PTV abutted the GI luminal structures, we cropped the PTV out the bowel and accepted underdosing of the GTV and PTV with no specific target volume criteria at those areas. Patients included in the study had fiducials placed before CT-simulation to assist with target delineation during treatment. Patients were treated to either 36 Gy in 3 fractions or 24 Gy in one fraction. The bowel was our major dose limiting structured and was limited to no more than 20 Gy (single fraction) and 30 Gy (multi-fraction) maximum dose. The max dose for the kidneys, liver, and cord were limited to 10 Gy, 20 Gy, 5 Gy (single fraction), and 15 Gy, 50 Gy, and 15 Gy (multi-fraction) respectively. Notably one patient exceeded the max dose for the left and right kidney (28.8 Gy and 29.0 Gy respectively).

### Endpoints

Our primary endpoints included overall survival (OS) from diagnosis and local-progression-free survival (LPFS) from SBRT. Secondary endpoints included regional-progression-free-survival (RPFS), distant-progression-free-survival (DPFS), and acute and late toxicity.

### Statistical analysis

Continuous variables were summarized with median and inter-quartile range (IQR). Categorical variables were summarized with frequency and percentage. The survival endpoints, LPFS, OS, RPFS, and DPFS, were analyzed with the Kaplan-Meier method. Patients were censored at last medical follow-up. The association of these survival endpoints with risk factors was studied with univariate Cox proportional hazards models. To build multivariable Cox models for the survival endpoints, the stepwise variable selection was performed. All the variables from univariate models that had a *p*-value of < 0.1 were included as potential predictors. Variables were subsequently removed from the multivariable model if the *p*-value was > 0.05. All *p*-values reported are two-sided. Acute toxicity was reported as crude rates occurring within 3 months of treatment. Late toxicity was considered toxicity that occurred >3 months following treatment. Actuarial late toxicity estimates were calculated by the Kaplan-Meier methods. The effect of factors on grade 2+ and grade 3+ toxicities were analyzed with logistic regression models.

## Results

### Patient characteristics

A detailed list of patient characteristics can be found in Table 1. We identified 145 patients with 146 lesions of pancreatic adenocarcinoma with a median age at diagnosis of 79 (range 70.1–90.3) with 55% female and 45% male. Tumors were most commonly located in the head (69%) of the pancreas. Nine patients (6%) had prior radiation with a median dose of 50.4 Gy (IQR, 50.4–55.8) in a median of 14.9 months prior to SBRT. Surgical stage at diagnosis, deemed in a multidisciplinary case review, included, resectable (30.3%), borderline resectable (15.9%), and unresectable (53.8%). Surgical resection was performed on 33.8% of the total patients. No patients with unresectable disease at diagnosis received a resection. Chemotherapy was given prior to (42.1%), concurrent with (4.1%) or follow SBRT (37.2%) Chemotherapy regimens included gemcitabine alone (45%), gemcitabine + capecitabine (29%), gemcitabine + other additional chemotherapy (20%), and FU based chemotherapy regimens (7%). Prior to SBRT, CA19-9 was elevated in 63.7%% (*n* = 93) of patients.

**Table 1:** Patient and treatment characteristics.

**Characteristics**	**Value (*n* = 145 patients)**
**Age (years, range)**	70.1–90.3
**Gender**	
Female	80 (55%)
Male	65 (45%)
**CA19-9 value (Median value, IQR)**	
At diagnosis	293.3 (101, 798)
Pre-SBRT	120.3 (34, 473.8)
Post-SBRT	85 (29.3, 436)
Change in CA19-9	−4.8 (−67.25, 79.95)
**Surgical stage**	
Resectable	44 (30.3%)
Borderline resectable	23 (15.9%)
Unresectable	78 (53.8%)
**Surgery**	
Yes	49 (33.8%)
No	96 (66.2%)
**Location**	
Body	14 (10%)
Head	100 (69%)
Tail	2 (1%)
Uncinate	9 (6%)
Neck	7 (5%)
Genu	1 (1%)
Multiple	12 (8%)
**Prior radiation (yes/no)**	
No	136 (94%)
Yes	9 (6%)
**Previous EBRT Dose (Median, IQR)**	50.4 (50.4, 55.8)
**Treatment platform**	
Trilogy	63 (43%)
CyberKnife	41 (28%)
Truebeam	41 (28%)
**Chemotherapy**	
Yes	103 (71.0%)
No	37 (24.8%)
Unavailable	9 (6.2%)
**Chemotherapy timing**	
Before SBRT	61 (42.1%)
Concurrent	5 (3.4%)
After SBRT	54 (37%)
**Chemotherapy agent**	
Gemcitibine	41 (45%)
Gemcitibine + Capcitabine	27 (29%)
Gemcitibine + other	18 (20%)
FU based	6 (7%)
**GTV (cm**^3^**) (Median, IQR)**	13 (8.7, 25)
**PTV (cm**^3^**) (Median, IQR)**	18 (12.6, 32)
**Fractionation**	
Single	54 (37%)
Multi-fraction	91 (63%)
**Dose (Median, IQR)**	36 (24, 36)

### SBRT treatment characteristics

SBRT was delivered by either Trilogy® (43%), Truebeam® (28%), or CyberKnife® (28%) in either one (37%) or multiple fractions (63%). Median BED_10_ and EQD_2_ were 81.6 (range 50.40–87.5) Gy and 68 (range 42–72.9) Gy (single fraction) and 79.2 (range 51.3–79.2) Gy and 66 (range 42.8–66) Gy (multi-fraction) respectively. Patients received SBRT as either definitive treatment (65.8%), or as neoadjuvant (8.3%) or adjuvant (25.5%) therapy in resected patients. Median dose was 36 Gy (IQR 24–36). For the entire cohort median gross tumor volume (GTV) was 13 cm^3^ (IQR 8.7–25) and planning target volume (PTV) was 18 cm^3^ (IQR 12.6–32). Following SBRT, CA19-9 levels were remeasured in a median of 1.9 months (IQR 1.2–2.9). Of the 93 patients with elevated baseline CA19-9 levels, 6 patients returned to normal levels following SBRT.

### Overall survival

Within a median follow-up of 12.3 months (IQR 6.0–23.3 months) the median survival from diagnosis for the entire cohort 14.0 months (95% CI: 12.3–16.5) with 1- and 2-year OS of 60% and 27.0%, respectively (Table 2). Median OS by resectability status was 24.4 months (95%CI: 15.6–33.1), 15.9 months (95%CI: 3.4–18.3), and 10.0 months (95%CI: 7.8–12.3) for resectable, borderline resectable, and unresectable, respectively. Univariate analysis demonstrated worse OS was significantly associated with increased age [*p* < 0.0081, HR 1.04 (95% CI, 1.01–1.070)], elevated pre-SBRT CA19-9 [*p* = 0.004, HR 1.00022 (95% CI, 1.00007–1.000360)], elevated post-SBRT CA19-9 [*p* < 0.001, HR 1.0004 (95% CI, 1.0002–1.00060)], increased stage [*p* < 0.001, HR 1.90 (95% CI, 1.52–2.37)], and increased PTV [*p* = 0.044, HR 1.01 (95% CI, 1.00–1.030)]. Improved OS was observed with recurrent lesions [*p* = 0.0173, HR 0.51 (95% CI, 0.30–0.89)], surgery [*p* < 0.001, HR 0.30 (95% CI, 0.20–0.45)], increased SBRT dose [*p* = 0.040, HR 0.97 (95% CI, 0.94–1.00)], and post-SBRT CA19-9 normalization [*p* = 0.045, HR 0.35 (95% CI, 0.12–0.980)]. On multivariate analysis, only surgery [*p* = 0.002, HR 0.36 (95% CI, 0.19–0.70)], post-SBRT CA19-9 normalization [*p* = 0.037, HR 0.32 (95%CI, 0.10–0.94)], and post-SBRT CA19-9 [*p* = 0.003, HR 1.0004 (95% CI, 1.0001–1.0005)] maintained significance on multivariate analysis (Table 3). Median survival for patients receiving resection was 28.3 months (95% CI: 16.2–40.5) vs. 11.4 months (95% CI: 9.3–13.5) in those without resection.

**Table 2:** Kaplan-Meier estimates for OS, LPFS, RPFS, and DPFS.

**Kaplan Meier-Estimates**	**All cohort**
**Median follow-up**	
From diagnosis—months (IQR)	12.3 (6.0–23.3)
**Median survival**	
Median survival—months (95% I)	40 (12.3–16.5)
12-months	60.0%
24-months	27.0 %
**Local control from SBRT**	
Median time to LF (95%CI)	Median not reached (20.8–infinity)
12-months	72%
24-months	63%
**Regional control from SBRT**	
Median time to RF (95% CI)	Median not reached (N/A)
12-months	92%
24-months	92%
**Distant metastases free survival**	
Median time to DM (95% CI)	23.1 (14.4–33.6)
12-months	62%
24-months	47%

**Table 3:** Results of univariate and multivariate cox regression models for OS.

**Factor**	**Hazard ratio (95% confidence interval)**	***p*-value**
**Univariate**	
Age	1.04 (1.01, 1.07)	0.008
CA19-9 at diagnosis	1.00003 (0.99999, 1.00006)	0.145
Pre-SBRT CA19-9	1.00022 (1.00007, 1.00036)	0.004
Post-SBRT CA19-9	1.0004 (1.0002, 1.0006)	<0.001
Change in CA19-9	1.00007 (0.99972, 1.00043)	0.687
CA19-9 normalization	0.35 (0.12, 0.98)	0.045
Previous EBRT Dose	0.03 (0.00, infinity)	0.998
Recurrent lesion vs. non-recurrent	0.51 (0.30, 0.89)	0.017
GTV volume (cm^3^)	1.004 (0.995, 1.013)	0.390
PTV volume (cm^3^)	1.01 (1.00, 1.03)	0.044
BED ≥ 60	0.77 (0.45, 1.30)	0.3268
Multiple fractions vs. single fraction	0.72 (0.51, 1.02)	0.068
Chemo: Gemcitibine + Capcitabine vs. Gemcitibine	0.92 (0.54, 1.55)	0.746
Chemo: FU based vs. Gemcitibine	0.51 (0.18, 1.44)	0.203
Stage	1.90 (1.52–2.37)	<0.001
Surgery vs. no surgery	0.30 (0.20, 0.45)	<0.001
Dose	0.97 (0.94, 1.00)	0.040
**Multivariate analysis**	
Surgery	0.36 (0.19, 0.70)	0.002
CA19-9 Normalization	0.32 (0.1, 0.94)	0.037
Post-SBRT CA19-9	1.0004 (1.0001, 1.0005)	0.003

### Local control

**Figure 1:**
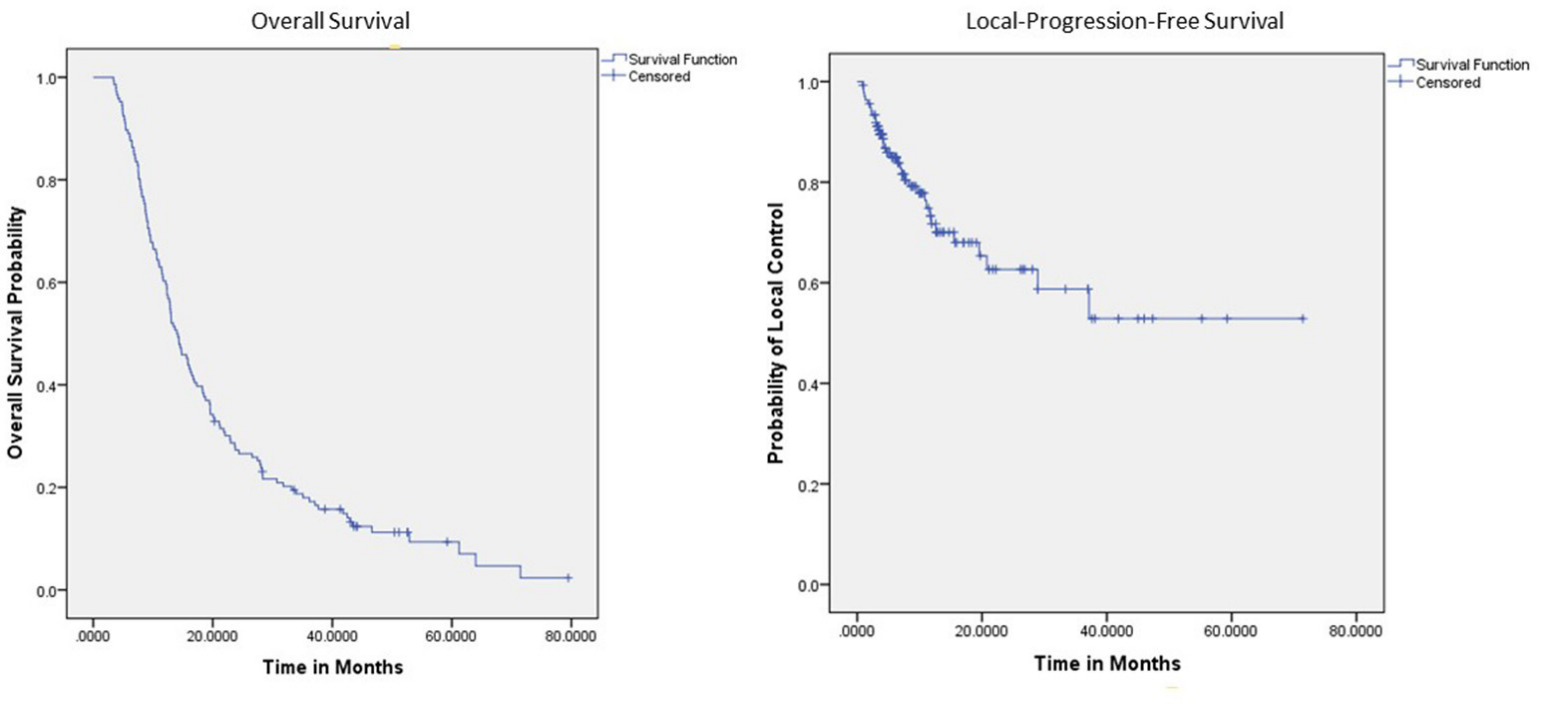
Kaplan Meier curves for OS and LPFS.

One- and 2-year LPFS is 72 and 63%, respectively for the entire cohort (Figure 1). Univariate analysis demonstrated significantly worse 2-year LPFS associated with elevated post-SBRT CA19-9 [*p* = 0.0216, HR 1.0038 (95% CI, 1.00006–1.00071)], and recurrent lesions [*p* < 0.005, HR 3.31 (95% CI, 1.42–7.68)]. Improved 2-year LPFS was observed with multi-fraction SBRT [*p* = 0.0189, HR 0.45 (95% CI, 0.23–0.88)]. On multivariate analysis, multi-fraction SBRT did not hold significance for superior local control. Only recurrent lesions [*p* = 0.0069, HR 5.1 (95% CI, 1.56–16.64)], and post-SBRT CA19-9 levels [*p* = 0.0107, HR 1.0005 (95% CI, 1.0001–1.0008)] maintained significance on multivariate analysis (Table 4). Within this cohort, 6.9% (*n* = 10) died of local progression.

**Table 4:** Results of univariate and multivariate Cox regression models for time to local progression.

**Factor**	**Hazard ratio (95% confidence interval)**	***p*-value**
**Univariate**	
Age	1.001 (0.946, 1.060)	0.962
CA19-9 at diagnosis	0.99997 (0.99974, 1.00019)	0.769
Pre-SBRT CA19-9	0.9996 (0.9986, 1.0005)	0.359
Post-SBRT CA19-9	1.00038 (1.00006, 1.00071)	0.022
Change in CA19-9	1.0003 (0.9998, 1.0008)	0.196
CA19-9 normalization	1.01 (0.28, 3.61)	0.9912
Previous EBRT Dose	0.01 (0.00, infinity)	1.000
Recurrent lesion vs. non-recurrent	3.31 (1.42, 7.68)	0.005
GTV volume	1.005 (0.988, 1.021)	0.570
PTV volume	1.01 (0.98, 1.03)	0.543
BED ≥ 60	2.09 (0.50, 8.73)	0.3136
Multiple fractions vs. single fraction	0.45 (0.23, 0.88)	0.019
Chemo: Gemcitibine + Capcitabine vs. Gemcitibine	0.51 (0.21, 1.24)	0.138
Chemo: FU based vs. Gemcitibine	0.62 (0.14, 2.72)	0.528
Stage	0.87 (0.59, 1.28)	0.45
Surgery vs. no surgery	0.83 (0.41, 1.69)	0.615
Dose	0.94 (0.89, 1.00)	0.049
**Multivariate**	
Recurrent lesion	5.10 (1.56, 16.64)	0.007
Post-SBRT CA19-9	1.0005 (1.0001, 1.0008)	0.011

### Regional and distant progression free survival

One- and 2-year RPFS rates were both 92%. None of the variables analyzed were found to be significantly associated with inferior regional control on univariate or multivariate analysis (Table 6s supplementary). At 1 and 2 years, the Kaplan-Meier estimated rate of DPFS was 62 and 47%, respectively. Univariate analysis identified elevated CA19-9 at diagnosis [*p* = 0.0006, HR 1.00015 (95% CI, 1.00006–1.00023)], and elevated pre-SBRT CA 19-9 [*p* = 0.0016, HR 1.00034 (95% CI, 1.00031–1.00056)] associated with inferior DPFS and surgery [*p* = 0.0493, HR 0.55 (95% CI, 0.31–1.00)] associated with superior DPFS (Table 7s in Supplementary Material). None of these variables maintained significance on multivariate analysis. Treatment fractionation was not found to be associated with either regional control or distant metastases.

### Radiation toxicity

For the entire cohort, 4.1 and 0.7% of patients experienced acute grade 2+ and 3+ toxicity respectively. Acute grade 2 toxicity included gastritis (*n* = 1), nausea (*n* = 2), and maculopapular rash (*n* = 2). One patient experienced acute grade 3 nausea requiring hospitalization. At 2-years late grade 2+ and 3+ toxicity was 2 and 1% respectively. One patient experienced a late grade 4 duodenal stenosis (8 months after SBRT) requiring urgent operative intervention. This patient was treated in 3 fractions and received a max dose to the small bowel of 25.5 Gy. This was likely a result of radiation and not tumor progression as there were no signs of local or regional progression. Two patients experienced late grade 3 toxicity which included nausea (*n* = 1) and enteritis (*n* = 1). None of the variables analyzed with univariate logistic regression were found to be significantly associated with acute or late grade 2+ or grade 3+ toxicity.

## Discussion

Pancreatic adenocarcinoma is primarily a malignancy of the elderly; however, this geriatric population is frequently underrepresented in clinic trials that aim to guide treatment decisions (5). As these patients are frequently more medically complex, special considerations need to be given when determining treatment planning. The dearth of evidence however makes this task especially challenging as the benefit of various treatment options is unclear. This retrospective review aimed to look for prognostic factors associated with outcomes for elderly patients treated with SBRT for pancreatic adenocarcinoma.

Our results did not identify any differences in overall survival, local, regional, or distant control, or toxicity between single or multi-fraction regimens. In contrast, our previous work looking at 289 patients (291 lesions) of all ages with pancreatic adenocarcinoma identified multi-fraction SBRT associated with improved local control on multivariate analysis [*p* = 0.009, HR 0.53 (95% CI, 0.33–0.85)] with a 2-year local control of 69.7 and 56.8% for multi-fraction and single fraction, respectively. It is possible that our lack of statistical significance on multivariate analysis is the smaller sampler size of the present study (*n* = 145) compared to our much larger previous report on all ages (*n* = 289).

Previously, Zhu et al. reported on outcomes of pancreatic cancer patients aged over 65 years treated with SBRT. They reported on 417 patients with advanced and medically inoperable pancreatic cancer with a median age of 73 years. Patients were treated with 30-46.8 Gy in 5–8 fractions. One-year OS, progression free survival (PFS), local-recurrence free survival (LRFS), and distant metastasis free survival (DMFS) were 35.5, 18.2, 26.6, and 27.1% respectively. Tumor stage, tumor response at 6 months, and CA19-9 level normalization at 3 months were all identified as predictors for OS, PFS, LRFS, and DMFS. Additionally, patients receiving 5-FU demonstrated improved survival compared to gemcitabine based chemotherapy. Finally, patients with BED_10_ ≥ 60 Gy achieved better tumor response as compared to those who received BED_10_ < 60 Gy (15). Compared to the present study, Zhu et al. reported worse outcomes for 1-year OS (35.5 vs. 60.0%), LRFS (26.6 vs. 72.0%), and DMFS (27.1 vs. 62%). These differences were likely a result of our report including patients with resectable disease. As such, 33.8% of patients in our cohort received surgical resection compared to 12.8% leading to the disparity in outcomes. Regarding prognostic factors, we also identified CA19-9 normalization to predict overall survival but not local control, regional control, or freedom from distant metastasis.

Unfortunately, as with, non-elderly patients, only 10–20% of patients are deemed to have resectable disease at the time of diagnosis (16–18). In addition to a large proportion of elderly patients having unresectable disease, elderly patients fare even worse as they are more likely to be medically-inoperable due to significant comorbidities. This has generated interest in radiation as definitive treatment for these patients. Specifically, SBRT has been the modality of choice for definitive treatment as its shorter duration allows patients to receive full dose systemic therapy with less delay (19). Burton et al. reported on 26 patients ≥ 80 years with pancreatic adenocarcinoma treated with definitive SBRT (24 Gy/ 1 fraction or 30-36 Gy/ 3 fractions) +/–chemotherapy. Median overall survival was 7.6 months with 34.6% 1-year survival and median local control was 11.5 months with 41.2% 1-year local control. This cohort exhibited no acute or late grade 3+ toxicity (20). The results of our study compare favorably with improved survival and local control however it is important to note our study included surgical patients which accounted for 34% (*n* = 49) of our cohort. Additionally, our cohort included younger patients than those above.

Compared to historical controls, our outcomes with SBRT appear to have a greater impact on resected patients than when used as definitive treatment. Prior reports have demonstrated a median OS of 16.1–23.4 months for resected patients treated with adjuvant chemotherapy alone or chemoradiation with EBRT (6, 7). Our results compare favorably with median OS of 28.1 months for resected patients treated with SBRT and chemotherapy. However, for patients with locally advanced disease, receiving either chemotherapy alone or chemoradiation with EBRT, median OS ranges from 8.6 to 11.4 months which is similar to our reported 11.4 months for patients receiving definitive SBRT (21–23).

The present study continues to support the prognostic role of CA19-9. Overall survival has been associated with both kinetic changes during chemotherapy as well as static values post operatively (24, 25). Additionally, as we have shown in our previous report of all ages, post-SBRT CA19-9 was associated with inferior overall survival and local control on multivariate analysis (26). Although numerous reports have demonstrated the prognostic importance of CA19-9 in the entire population, few studies have assessed if this holds true for elderly patients. Frakes et al. assessed pancreatic cancer outcomes in the elderly and found post-operative CA19-9 greater than 90 (*p* < 0.001, HR 2.81) was associated with worse survival (6).

The present study also identified recurrent pancreatic adenocarcinoma significantly associated with inferior local control. Treatment of recurrent pancreatic cancer has been challenging due to limited therapeutic options (27, 28). Surgical re-resection, EBRT, SBRT, and systemic chemotherapy have all been used and have their limitations (29–32). The precision SBRT provides is especially useful in recurrent patients as they have often received prior radiation therapy. Previous reports have identified SBRT to be a safe and reasonable treatment option for locally recurrent pancreatic cancer capable of providing symptoms palliation (33, 34). Within our cohort, recurrent patients were often treated with reirradiation, and it is therefore possible that their SBRT treatment plan was more conservative to reduce potential toxicity. This could have led to the observed inferior local control compared to non-recurrent tumors. Additionally, it is possible that recurrent tumors are more locally aggressive and therefore likely to re-recur.

Here we add to currently limited literature of pancreatic cancer treatment in elderly patients. This identified no differences in outcomes or toxicity between single fraction and multi-fraction SBRT for this cohort. Further, we confirmed SBRT for pancreatic adenocarcinoma in elderly patients to be a safe and effective treatment modality for this challenging population with very low rates of toxicity. This study however was limited by its retrospective nature. Firstly, patients were treated on three different treatment platforms and received a non-standardized treatment regimen. The patients included in this study also represented a heterogeneous population including resectable, borderline resectable, and unresectable patients that received various surgical and chemotherapeutic treatments. Additionally, we did not have any object data regarding pain control or symptom palliation following treatment which represents a critically important aspect among elderly patients. Finally, our toxicity may be underrepresented due to poor follow-up with radiation oncology. Prospective studies will be needed for a more rigorous assessment of the role of fractionation on patient outcomes and the role of SBRT in the elderly more generally.

## Conclusion

Surgical resection, post-SBRT CA 19-9, and post-SBRT CA 19-9 normalization appears to be predictive of clinical outcomes in elderly patients with pancreatic adenocarcinomas. SBRT can be delivered with minimal acute and late toxicity and therefore little impact on patients' quality of life. Future studies should further refine treatments based on these characteristics.

## Author contributions

PS: data collection, data analysis, wrote, and prepared manuscript; MB and DH: development of project, review and editing of final manuscript; Provided insight into how results relate to radiation oncology. HW: data analysis, ran all statistical tests, review, and editing of final manuscript.

### Conflict of interest statement

The authors declare that the research was conducted in the absence of any commercial or financial relationships that could be construed as a potential conflict of interest.
