# Supplementary material for: Prognostic Factors for Elderly Patients Treated With Stereotactic Body Radiation Therapy for Pancreatic Adenocarcinoma
Source: Front Oncol. 2018 Jul 27;8:282. doi: 10.3389/fonc.2018.00282 (PMC6072866; doi:10.3389/fonc.2018.00282)
Supplement: Supplementary file 1 [file Table_6.docx]

**Supplementary Materials**

Table 6s: Results of univariate and multivariate Cox regression models for time to regional progression.

| **Factor** | **Hazard ration (95% confidence interval)** | **p-value** |
| --- | --- | --- |
| Univariate |  |  |
| Age | 0.97 (0.87, 1.08) | 0.530 |
| CA19-9 at diagnosis | 1.00009 (0.99990, 1.00028) | 0.368 |
| Pre-SBRT CA19-9 | 1.0001 (0.9996, 1.0006) | 0.662 |
| Post-SBRT CA19-9 | 1.0004 (0.9999, 1.0009) | 0.113 |
| Change in CA19-9 | 1.0001 (0.9992, 1.0011) | 0.778 |
| CA19-9 Normalization | 1.60 (0.12, 20.78) | 0.7187 |
| SMAD4 mutated vs not | 1.00 (0.28, 3.57) | 0.997 |
| Location: Head vs Body | 0.73 (0.09, 6.12) | 0.774 |
| Previous EBRT Dose | NA (there were only 6 patients with dose data, and they all had no regional progression) | NA |
| Modality: CyberKnife vs Triology | 0.51 (0.11, 2.39) | 0.389 |
| Modality: Truebeam vs Triology | 0.00 (0.00, infinity)* | 0.994 |
| Recurrent lesion being treated | 0.00 (0.00, infinity)* | 0.994 |
| GTV volume | 1.02 (1.00, 1.04) | 0.118 |
| PTV volume | 1.02 (0.98, 1.06) | 0.265 |
| BED ≥ 60 | 3949730.28 (0.00, infinity) | 0.9939 |
| Multiple fractions | 1.07 (0.28, 4.14) | 0.924 |
| Chemo: Gemcitibine+Capcitabine vs Gemcitibine | 0.53 (0.10, 2.76) | 0.454 |
| Chemo: Gemcitibine+other vs Gemcitibine | 0.44 (0.05, 3.78) | 0.454 |
| Chemo: FU based vs Gemcitibine | 1.08 (0.13, 9.27) | 0.945 |
| Stage | 1.01 (0.58-1.75) | 0.97 |
| Surgery | 0.21 (0.04, 1.12) | 0.068 |
| Dose | 1.02 (0.91, 1.15) | 0.699 |

*The hazard ratio is 0 because the group defined by value 1 of the variable did not have a regional progression event.

No variables reached significance on multivariate analysis

Table 7s: Results of univariate and multivariate Cox regression models for time to distant progression.

| **Factor** | **Hazard ration (95% confidence interval)** | **p-value** |
| --- | --- | --- |
| age | 0.98 (0.94, 1.02) | 0.365 |
| CA19-9 at diagnosis | 1.00015 (1.00006, 1.00023) | <.001 |
| Pre-SBRT CA19-9 | 1.00034 (1.00013, 1.00056) | 0.002 |
| Post-SBRT CA19-9 | 1.00008 (0.99979, 1.00038) | 0.572 |
| Change in CA19-9 | 0.9998 (0.9994, 1.0002) | 0.327 |
| CA19-9 Normalization | 0.45 (0.10, 1.98) | 0.2919 |
| SMAD4 mutated vs not | 0.79 (0.50, 1.23) | 0.292 |
| Location: Head vs Body | 0.58 (0.26, 1.32) | 0.195 |
| Previous EBRT Dose | 0.03 (0.00, infinity) | 0.999 |
| Modality: CyberKnife vs Trilogy | 1.35 (0.69, 2.63) | 0.379 |
| Modality: Truebeam vs Trilogy | 1.48 (0.79, 2.78) | 0.223 |
| Recurrent lesion being treated | 0.47 (0.14, 1.50) | 0.200 |
| GTV volume | 1.00 (0.99, 1.02) | 0.986 |
| PTV volume | 1.01 (0.99, 1.03) | 0.204 |
| BED ≥60 | 1.15 (0.45, 2.90) | 0.7735 |
| Multiple fractions | 0.88 (0.50, 1.54) | 0.652 |
| Chemo: Gemcitibine+Capcitabine vs Gemcitibine | 1.31 (0.65, 2.67) | 0.448 |
| Chemo: Gemcitibine+other vs Gemcitibine | 1.12 (0.48, 2.62) | 0.797 |
| Chemo: FU based vs Gemcitibine | 0.99 (0.29, 3.44) | 0.991 |
| Stage | 1.01 (0.81-1.27) | 0.91 |
| Surgery | 0.55 (0.31, 1.00) | 0.049 |
| Dose | 0.99 (0.95, 1.04) | 0.778 |

*The hazard ratio is 0 because the group defined by value 1 of the variable did not have a distant progression event.

No variables reached significance on multivariate analysis
